# Supplementary material for: The deubiquitylase Ubp15 couples transcription to mRNA export
Source: eLife. 2020 Nov 23;9:e61264. doi: 10.7554/eLife.61264 (PMC7682988; doi:10.7554/eLife.61264)
Supplement: Transparent reporting form [file elife-61264-transrepform.docx]

***eLife’s* transparent reporting form**

We encourage authors to provide detailed information *within their submission* to facilitate the interpretation and replication of experiments. Authors can upload supporting documentation to indicate the use of appropriate reporting guidelines for health-related research (see [EQUATOR Network](about:blank)), life science research (see the BioSharing Information Resource), or the [ARRIVE guidelines](about:blank) for reporting work involving animal research. Where applicable, authors should refer to any relevant reporting standards documents in this form.

If you have any questions, please consult our Journal Policies and/or contact us: [editorial@elifesciences.org](about:blank).

**Sample-size estimation**

- You should state whether an appropriate sample size was computed when the study was being designed
- You should state the statistical method of sample size computation and any required assumptions
- If no explicit power analysis was used, you should describe how you decided what sample (replicate) size (number) to use

Please outline where this information can be found within the submission (e.g., sections or figure legends), or explain why this information doesn’t apply to your submission:

Experiments were performed using biological replicates, the number of which being as typically done in the field (typically 2 to 3 biological replicates; see next section for details). Biological replicate numbers are as follow:

All this information is stated in the Materials and Methods section.

**Replicates**

- You should report how often each experiment was performed
- You should include a definition of biological versus technical replication
- The data obtained should be provided and sufficient information should be provided to indicate the number of independent biological and/or technical replicates
- If you encountered any outliers, you should describe how these were handled
- Criteria for exclusion/inclusion of data should be clearly stated
- High-throughput sequence data should be uploaded before submission, with a private link for reviewers provided (these are available from both GEO and ArrayExpress)

Please outline where this information can be found within the submission (e.g., sections or figure legends), or explain why this information doesn’t apply to your submission:

All experiments were performed in biological replicates (each starting from an individual yeast colony) as follow:

- ChIP-qPCR (3);

- ChIP-chip (2);

- Serial-dilution growth assays (at least 2, unless otherwise mentioned, performed independently on separate days and sometimes repeated in different genetic backgrounds);

- Affinity purification followed by MS (2 for RNAPII, 1 for Ubp15 although we also repeated this experiment in different conditions (different salt concentration (data not shown)), 3 for Mex67);

- RNA FISH (Experiment was performed several times to optimize the technique and the data shown in the paper is for one experiment with optimized conditions where we counted at least 150 cells from each strain);

- In vivo ubiquitylation assay (3 for experiments from Figure 5; 2 for experiments from Figure 5-supplement 1, except for Nup133, which was assayed only once)

Note that for ChIP-qPCR each biological replicate was measured in technical triplicates by qPCR (3 separate qPCR reactions from the same biological sample).

All this information is stated in the Materials and Methods section.

Microarray data and processed files have been deposited in GEO under the accession number GSE154671. Mass spectrometry data have been deposited in MassIVE under accession numbers MSV000085729, MSV000085730 and MSV000085731. Private links were provided to reviewers.

**Statistical reporting**

- Statistical analysis methods should be described and justified
- Raw data should be presented in figures whenever informative to do so (typically when N per group is less than 10)
- For each experiment, you should identify the statistical tests used, exact values of N, definitions of center, methods of multiple test correction, and dispersion and precision measures (e.g., mean, median, SD, SEM, confidence intervals; and, for the major substantive results, a measure of effect size (e.g., Pearson's r, Cohen's d)
- Report exact p-values wherever possible alongside the summary statistics and 95% confidence intervals. These should be reported for all key questions and not only when the p-value is less than 0.05.

Please outline where this information can be found within the submission (e.g., sections or figure legends), or explain why this information doesn’t apply to your submission:

In Figure 5D a volcano plot is shown where the p-value was obtained from a T-test on the spectral counts of the three WT and three ubp15Δ samples. This information is stated in the Materials and Methods section.

The number of genes (N) used in Figures 3B and Figure 3-supplement 1 are indicated in their legends.

In Figure 4D, the number of cells counted for each strain is indicated in the legend.

(For large datasets, or papers with a very large number of statistical tests, you may upload a single table file with tests, Ns, etc., with reference to sections in the manuscript.)

**Group allocation**

- Indicate how samples were allocated into experimental groups (in the case of clinical studies, please specify allocation to treatment method); if randomization was used, please also state if restricted randomization was applied
- Indicate if masking was used during group allocation, data collection and/or data analysis

Please outline where this information can be found within the submission (e.g., sections or figure legends), or explain why this information doesn’t apply to your submission:

Not applicable

**Additional data files (“source data”)**

- We encourage you to upload relevant additional data files, such as numerical data that are represented as a graph in a figure, or as a summary table
- Where provided, these should be in the most useful format, and they can be uploaded as “Source data” files linked to a main figure or table
- Include model definition files including the full list of parameters used
- Include code used for data analysis (e.g., R, MatLab)
- Avoid stating that data files are “available upon request”

Please indicate the figures or tables for which source data files have been provided:

We provided 4 supplementary files as follow:

Supplementary file 1. List of the proteins associated with RNAPII and their differential association in fcp1-1 cells. Related to Figure 1B.

Supplementary file 2. List of the proteins associated with Mex67 and their differential association in ubp15D cells. Related to Figure 5D.

Supplementary file 3. List of yeast strains used in this study.

Supplementary file 4. List of plasmids used in this study.
